# Mass Cytometry Reveals Comparable Proportions of Leukocyte Subsets and Cell Surface P2X7 or CD39 on Human Peripheral Blood Mononuclear Cell Samples Isolated by Ficoll-Paque or SepMate Tube Density Gradient Centrifugation

**DOI:** 10.3390/cells15090795

**Published:** 2026-04-27

**Authors:** Amal Elhage, Bavani Gunasegaran, Natalie Smith, Ross J. Turner, Debbie Watson, Helen M. McGuire, Ronald Sluyter

**Affiliations:** 1 Molecular Horizons and School of Science, University of Wollongong, Wollongong, NSW 2522, Australia; aelhage@uow.edu.au (A.E.); ross.turner@sydney.edu.au (R.J.T.); dwatson@uow.edu.au (D.W.); 2 Charles Perkins Centre, School of Medical Sciences, Faculty of Medicine and Health, The University of Sydney, Sydney, NSW 2006, Australia; bavani.gunasegaran@nccs.com.sg (B.G.); natalie.smith1@sydney.edu.au (N.S.); helen.mcguire@sydney.edu.au (H.M.M.); 3 Program in Translational and Clinical Liver Cancer Research, Division of Medical Science, National Cancer Center Singapore, Singapore 168583, Singapore; 4 Sydney Cytometry Core Research Facility, The University of Sydney and Centenary Institute, Sydney, NSW 2006, Australia

**Keywords:** lymphocyte, monocyte, dendritic cell, *P2RX7*, *ENTPD1*, NTPDase1, cell separation, mass cytometry

## Abstract

**Highlights:**

**What are the main findings?**

- Proportions of leukocyte subsets are similar in human PBMC samples isolated by Ficoll-Paque or SepMate Tube density centrifugation.
- Cell surface P2X7 or CD39 on leukocyte subsets are similar in human PBMC samples isolated by Ficoll-Paque or SepMate Tube density centrifugation.

**What are the implications of the main findings?**

- SepMate Tube density centrifugation is a suitable alternative to Ficoll-Paque density centrifugation for the isolation of human PBMC samples in biobanking.
- SepMate Tube density centrifugation is a suitable alternative to Ficoll-Paque density centrifugation for the study of human PBMC subsets and cell surface purinergic molecules by mass cytometry.

**Abstract:**

P2X7 is an adenosine 5′-triphosphate (ATP)-gated ion channel. CD39 (ectonucleotidase triphosphate diphosphohydrolase-1) hydrolyses ATP to reduce its extracellular concentration to limit P2X7 activation. Both P2X7 and CD39 are present on leukocytes. Ficoll-Paque density gradient centrifugation is widely used to isolate human peripheral blood mononuclear cells (PBMCs), yet the extent to which other density gradient centrifugation methods influence the expression of cell surface molecules remains unclear. Using mass cytometry, this study compared the proportions of mononuclear leukocyte subsets and the relative amount of cell surface P2X7 and CD39 detected on these cells in paired, bar-coded cryopreserved human PBMC samples isolated by Ficoll-Paque or SepMate Tube density gradient centrifugation. Both techniques yielded similar proportions of mononuclear leukocyte subsets. P2X7 and CD39 were detected across all cell subsets, with the relative amount of P2X7 or CD39 comparable between separation methods. Relatively minor but statistically significant differences were observed for some populations. P2X7 expression was higher on CD3^+^, CD4^+^, and conventional CD4^+^ T cells, and naïve B cells, and lower on myeloid dendritic cells, while CD39 expression was lower on regulatory T cells in SepMate Tube samples compared to Ficoll-Paque samples. Overall, Ficoll-Paque and SepMate Tube density gradient centrifugation yield comparable results within PBMC samples, supporting the use of either method in studies examining immune phenotypes including the purinergic molecules P2X7 and CD39.

## 1. Introduction

P2X7 is a ligand-gated ion channel belonging to the purinergic type 2 receptor family of the purinergic signalling system [1]. P2X7 is activated by extracellular adenosine 5′-triphosphate (ATP) and is present on leukocytes including dendritic cells (DCs), macrophages, monocytes, and innate and adaptive lymphocytes [2]. Activation of P2X7 on these cell types induces an array of downstream events including the release of soluble mediators, and cell differentiation, proliferation, and death [3]. As such, P2X7 plays important roles in inflammation and immunity and related disorders [2], including infectious diseases [4] and cancers [5]. Therefore, P2X7 represents a potential therapeutic target and biomarker in a range of disorders [6].

CD39 (ectonucleoside triphosphate diphosphohydrolase-1) is an ectonucleotidase which hydrolyses extracellular ATP to adenosine 5′-diphosphate (ADP), and ADP to adenosine 5′-monophosphate [7]. CD39 is present on various leukocyte subsets including DCs, macrophages, monocytes, natural killer (NK) cells, B cells, and regulatory T cells (Tregs) [8]. The hydrolysis of extracellular ATP by CD39 has been shown to limit P2X7 activation on various cell types including DCs [9], macrophages [10,11], and mast cells [12]. Thus, CD39 can be considered a negative regulator of P2X7-mediated processes [12] and like P2X7 represents a potential therapeutic target and biomarker in a range of disorders [13]. Notably, various studies have reported the presence of CD39 on various subsets of human peripheral blood mononuclear cells (PBMCs) as a biomarker in a number of disorders including systemic lupus erythematosus [14], multiple sclerosis [15], head and neck cancer [16], and COVID-19 [17].

Isolation of human PBMCs by Ficoll-Paque density gradient centrifugation dates back some 50 years [18,19]. However, this method requires careful handling and prolonged centrifugation to prevent layer mixing, prompting several modern refinements to this approach for easier and faster isolation. One example is the use of SepMate™ Tubes, introduced by STEMCELL Technologies (Vancouver, BC, Canada), which includes an insert for quicker preparation. STEMCELL Technologies also offers Lymphoprep™ density gradient medium, an alternative to Ficoll-Paque, which is added to SepMate Tubes. Despite the use of these isolation procedures in many biobanks, studies comparing PBMCs isolated by SepMate Tube density gradient centrifugation to those isolated by Ficoll-Paque density gradient centrifugation are limited, and it remains largely unknown if these isolation procedures alter the expression of cell surface purinergic molecules [20,21]. Therefore, using mass cytometry this study aimed to compare the proportions of mononuclear leukocyte subsets in PBMCs isolated by Ficoll-Paque or SepMate Tube density gradient centrifugation, and the relative expression of P2X7 and CD39 on these subsets.

## 2. Materials and Methods

### 2.1. PBMC Isolation by Ficoll-Paque or SepMate Tube Density Gradient Centrifugation

Whole blood was collected into Vacutainer heparin tubes (BD Biosciences, San Diego, CA, USA) from healthy human donors (five males; age range 21–27 years) with informed consent, and was studied in accordance with the approvals provided by the University of Wollongong Human Ethics Committee (12/219 and 19/100). Blood samples from each subject were divided and used for the isolation of PBMCs by Ficoll-Paque or SepMate Tube density gradient centrifugation within 4 h of collection.

PBMCs were isolated by Ficoll-Paque density gradient centrifugation as described [21]. Approximately 16 mL of whole blood in 50 mL conical bottom tubes was diluted in an equal volume of room temperature (RT) sterile Dulbecco’s phosphate-buffered saline (PBS) (Gibco, Life Technologies, Paisley, UK). Diluted samples were then underlaid with Ficoll-Paque™ Plus (GE Healthcare, Uppsala, Sweden) and centrifuged (600× *g* for 30 min with the brake disengaged at RT). PBMCs were recovered from the gradient interface, transferred to new 50 mL conical bottom tubes and were washed with RPMI-1640 medium (Gibco, Life Technologies, Grand Island, NY, USA) containing 10% foetal calf serum (FCS) (Bovogen Biologicals, Kelor East, Australia) (RPMI-FCS) (500× *g* for 10 min at 4 °C).

PBMCs were isolated by SepMate Tube density gradient centrifugation as described [21]. Briefly, RT Lymphoprep (STEMCELL Technologies) was pipetted into SepMate Tubes (STEMCELL Technologies). Approximately 16 mL of whole blood in 50 mL conical bottom tubes was diluted in an equal volume of RT sterile PBS before being carefully transferred by pipetting down the side of the SepMate Tube. Tubes were then centrifuged (1200× *g* for 10 min at RT). Plasma and PBMCs were poured into new 50 mL conical-bottom tubes and washed in RPMI-FCS (500× *g* for 10 min at 4 °C).

### 2.2. Cryopreservation of PBMCs

Isolated PBMCs were cryopreserved as described [21]. Briefly, PBMCs in RPMI-FCS were centrifuged (400× *g* for 10 min at 4 °C) and resuspended in RPMI-FCS at 6–10 × 10^6^ cells/mL. Cells were then diluted with an equal volume of ice-cold freeze down medium [RPMI-1640 medium containing 40% FCS and 20% dimethyl sulfoxide (Sigma-Aldrich, St Louis, MO, USA)] in a dropwise fashion. Cells were placed overnight in a Nalgene Cryo 1 °C Freezing Container (Nalgene Nunc, Rochester, NY, USA) containing 100% isopropanol, then transferred to liquid nitrogen until required.

### 2.3. Immunolabelling and Mass Cytometry of PBMCs

**Figure 1:**
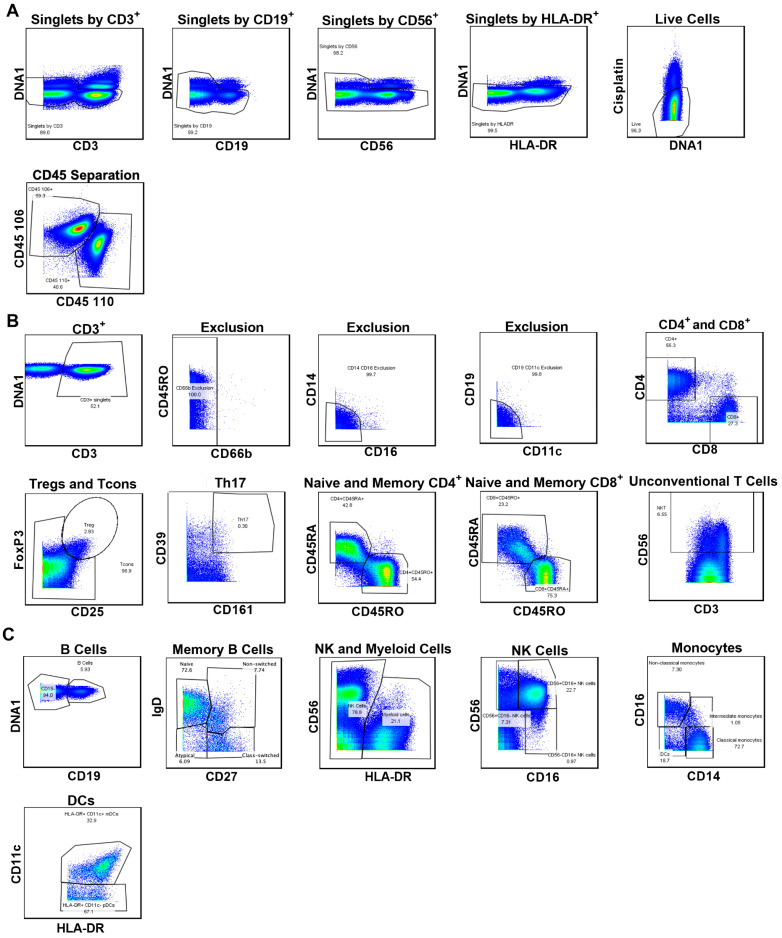
Gating strategy used to identify human mononuclear leukocyte subsets. (**A**–**C**) PBMCs were labelled with metal-conjugated mAbs and analysed by mass cytometry. (**A**) Starting with non-EQ bead acquired events, total singlets were sequentially gated by using DNA intercalated (DNA1) with CD3^+^, CD19^+^, CD56^+^, and HLA-DR^+^. Live cells were gated on DNA1 and cisplatin. Cells were then gated on differentially barcoded anti-CD45 mAb. (**B**) From CD45^+^ cells, CD3^+^ T cells were gated and CD66b^+^, CD16^+^CD14^+^, and CD11c^+^CD19^+^ non-T cell events were excluded, before gating CD4^+^ and CD8^+^ T cells, CD25^+^FoxP3^+^ Tregs, CD25^−/+^FoxP3^−^ Tcons, CD4^+^CD161^+^CD39^+^ Th17 cells, CD4^+^CD45RO^+^ memory T cells, CD4^+^CD45RA^+^ naïve T cells, CD8^+^CD45RO^+^ memory T cells, CD8^+^CD45RA^+^ naïve T cells, and CD3^+^CD56^+^ unconventional T cells. (**C**) From CD45^+^ cells, CD19^+^ B cells, from CD19^+^ B cells CD27^−^IgD^+^ naïve B cells, or CD27^+^IgD^+^ non-switched, CD27^+^IgD^−^ class-switched, or CD27^−^IgD^−^ atypical memory B cells; from CD45^+^ cells HLA-DR^+^ myeloid cells and CD56^+/−^CD16^+/−^ NK cells, CD14^+/−^CD16^+/−^ monocytes and HLA-DR^+^CD11c^+^ mDCs and HLA-DR^+^CD11c^−^ pDCs were gated. Flow cytometric plots are representative of a PBMC sample isolated by Ficoll-Paque density centrifugation.

Immunolabelling and mass cytometry was performed as described [22]. Cryopreserved PBMC samples were thawed at 37 °C and washed with warm RPMI-FCS and 1:10,000 Pierce Universal Nuclease (Thermo Fisher Scientific, Waltham, MA, USA) (350× *g* for 8 min). Cells were washed in serum-free RPMI-1640 medium (500× *g* for 5 min). Cells in 5 mL round-bottom tubes from paired samples were initially incubated with anti-CD45 monoclonal antibodies (mAbs) conjugated to ^106^Pd (Ficoll-Paque) or ^110^Pd (SepMate Tube) (Table 1) for 30 min on ice, for barcoding [23] (Figure 1A ‘CD45 Separation’). Then, 1 mL of FACS buffer (0.5% bovine serum albumin, 0.02% sodium azide, and 2 mM ethylenediaminetetraacetic acid, in PBS) was added to each tube. Paired samples of Ficoll-Paque and SepMate Tube isolated PBMCs were combined into one tube and centrifuged (500× *g* for 5 min). Cells were stained with 1.25 µM cisplatin (Standard BioTools, South San Francisco, CA, USA), to exclude dead cells, for 3 min on ice. Cells were then washed once with RPMI-FCS, then twice with FACS buffer (500× *g* for 5 min). Cells were incubated with a cocktail of metal-conjugated mAbs (Table 1) for 30 min on ice. Cells were washed with FACS buffer (500× *g* for 5 min) before being fixed and permeabilised using the FoxP3/Transcription Factor Staining Buffer Set (eBioscience, San Diego, CA, USA) according to the manufacturer’s instructions. Cells were incubated with an anti-Forkhead box protein P3 (FoxP3) mAb for 45 min in the dark on ice. Cells were washed and fixed overnight in 4% paraformaldehyde containing DNA intercalator (0.125 mM ^191/193^Ir, Standard BioTools). Cells were washed once with FACS buffer, then ultrapure water, and finally MaxPar Cell Acquisition Solution (CAS) (Standard BioTools) (900× *g* for 8 min). Cells were diluted to 800,000 cells/mL in CAS containing 1:10 diluted EQ beads (Standard BioTools) and filtered through a 35 μm nylon cell stainer snap-cap (Falcon, Corning, New York, NY, USA). Cells were acquired using a Standard BioTools CyTOF 2 Helios upgraded mass cytometer. Data were normalised with the concurrently run EQ Beads using the CyTOF acquisition software and subsequently analysed using FlowJo software v10.7.1 (BD Biosciences, Franklin Lakes, NJ, USA). Mean metal intensity (MMI) of mAb labelling was determined using the geometric mean function of FlowJo software.

### 2.4. Data Presentation and Statistics

Data is shown for five individual donors. Data were tested for normality using a Shapiro–Wilk test. Statistical differences were calculated using a paired Student’s *t*-test (parametric) or Wilcoxon test (non-parametric). The percentage change (%Δ) in each immune cell subset was calculated for each donor by calculating the difference between SepMate Tube values to Ficoll-Paque values (100%). The %Δ values displayed above each panel represent the mean %Δ across all donors ± standard error of the mean. All statistical analyses were conducted, and graphs were assembled, using GraphPad Prism software v8.0.2 (GraphPad Software; La Jolla, CA, USA). For all analyses, differences were considered significant if *p* < 0.05.

## 3. Results

### 3.1. Ficoll-Paque and SepMate Tube Density Gradient Separation of PBMCs Yield Similar Proportions of Mononuclear Leukocyte Subsets

**Figure 2:**
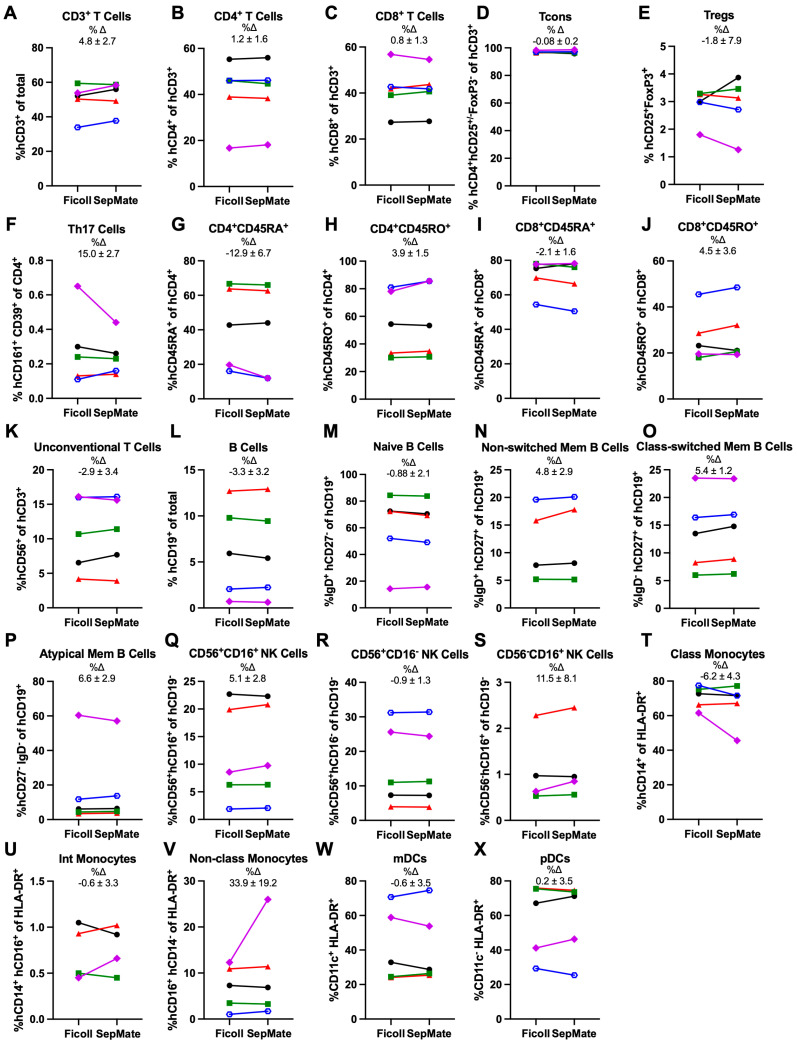
Ficoll-Paque and SepMate Tube density gradient centrifugation of PBMCs yield similar proportions of mononuclear leukocyte subsets. (**A**–**X**) Paired PBMC samples, isolated by Ficoll-Paque (Ficoll) or SepMate Tube (SepMate) density gradient centrifugation, were labelled with metal-conjugated mAbs and analysed by mass cytometry. Proportions, as mean %Δ ± SEM in SepMate PBMCs compared to Ficoll PBMCs, of (**A**) CD3^+^ T cells, (**B**) CD4^+^ T cells, (**C**) CD8^+^ T cells, (**D**) CD25^−/+^FoxP3^−^ Tcons, (**E**) CD25^+^FoxP3^+^ Tregs, (**F**) CD4^+^CD161^+^CD39^+^ Th17 cells, (**G**) naïve CD4^+^CD45RA^+^ T cells, (**H**) memory CD4^+^CD45RO^+^ T cells, (**I**) naïve CD8^+^CD45RA^+^ T cells, (**J**) memory CD8^+^CD45RO^+^ T cells, (**K**) CD3^+^CD56^+^ unconventional T cells, (**L**) CD19^+^ B cells, (**M**) CD27^−^IgD^+^ naïve B cells, (**N**) CD27^+^IgD^+^ non-switched memory B cells, (**O**) CD27^+^IgD^−^ class-switched memory B cells, (**P**) CD27^−^IgD^−^ atypical memory B cells, (**Q**) CD56^+^CD16^+^ NK cells, (**R**) CD56^+^CD16^−^ NK cells, (**S**) CD56^−^CD16^+^ NK cells, (**T**) HLA-DR^+^CD14^+^CD16^−^ classical monocytes, (**U**) HLA-DR^+^CD14^+^CD16^+^ intermediate monocytes, (**V**) HLA-DR^+^CD14^−^CD16^+^ non-classical monocytes, (**W**) HLA-DR^+^CD11c^+^ mDCs, and (**X**) HLA-DR^+^CD11c^−^ pDCs. Symbols represent individual donors (*n* = 5 donors). Statistical analyses were conducted using a (**A**–**D**, **F**–**J**, **L**–**O**, **Q**–**X**), paired Student’s *t*-test, or (**E**,**K**,**P**) Wilcoxon test.

To determine if there were differences between proportions of mononuclear leukocyte subsets in PBMCs isolated by Ficoll-Paque or SepMate Tube density gradient centrifugation, cells were incubated with metal-conjugated mAbs (Table 1) and then analysed by mass cytometry using a consistent gating strategy (Figure 1). All subsets analysed were present in PBMCs isolated by either separation technique (Figure 2).

Analysis of T cell subsets (as mean %Δ ± SEM of SepMate isolated PBMCs compared to Ficoll-Paque isolated PBMCs) showed that proportions of CD3^+^ T cells (%Δ 4.8 ± 2.7) (*p* = 0.16) (Figure 2A), total CD4^+^ T cells (%Δ 1.2 ± 1.6) (*p* = 0.87) (Figure 2B), total CD8^+^ T cells (%Δ 0.8 ± 1.3) (*p* = 0.86) (Figure 2C), conventional T cells (Tcons) (%Δ −0.08 ± 0.2) (*p* = 0.80) (Figure 2D), Tregs (%Δ −1.8 ± 7.9) (*p* = 1.0) (Figure 2E), and T helper (Th)17 cells (%Δ 15.0 ± 2.7) (*p* = 0.42) (Figure 2F) were comparable between separation techniques. Likewise, proportions of naïve CD4^+^CD45RA^+^ T cells (%Δ −12.9 ± 6.7) (*p* = 0.18) (Figure 2G), memory CD4^+^CD45RO^+^ T cells (%Δ 3.9 ± 1.5) (*p* = 0.16) (Figure 2H), naïve CD8^+^CD45RA^+^ T cells (%Δ −2.1 ± 1.6) (*p* = 0.38) (Figure 2I), memory CD8^+^CD45RO^+^ T cells (%Δ 4.5 ± 3.6) (*p* = 0.28) (Figure 2J), and unconventional T cells (%Δ −2.9 ± 3.4) (*p* = 0.49) (Figure 2K), which comprise a mixture of innate T cells [27], were comparable between separation techniques.

In regard to B cell types, proportions of CD19^+^ B cells (%Δ −3.3 ± 3.2) (*p* = 0.44) (Figure 2L), naïve B cells (%Δ −0.88 ± 2.1) (*p* = 0.15) (Figure 2M), non-switched memory B cells (%Δ 4.8 ± 2.9) (*p* = 0.21) (Figure 2N), class-switched B cells (%Δ 5.4 ± 1.2) (*p* = 0.09) (Figure 2O), and atypical memory B cells (%Δ 6.6 ± 2.9) (*p* = 0.63) (Figure 2P) were comparable between separation techniques.

In regard to other leukocytes, CD56^+^CD16^+^ NK cells (%Δ 5.1 ± 2.8) (*p* = 0.27) (Figure 2Q), CD56^+^CD16^−^ NK cells (%Δ −0.9 ± 1.3) (*p* = 0.57) (Figure 2R), CD56^−^CD16^+^ NK cells (%Δ 11.5 ± 8.1) (*p* = 0.18) (Figure 2S), classical monocytes (%Δ −6.2 ± 4.3) (*p* = 0.29) (Figure 2T), intermediate monocytes (%Δ −0.6 ± 3.3) (*p* = 0.77) (Figure 2U) and non-classical monocytes (%Δ 33.9 ± 19.2) (*p* = 0.35) (Figure 2V), myeloid (m) DCs (%Δ −0.6 ± 3.5) (*p* = 0.82) (Figure 2W), and plasmacytoid (p) DCs (%Δ 0.2 ± 3.5) (*p* = 0.82) (Figure 2X) were also comparable between separation techniques.

### 3.2. Cell Surface P2X7 Expression Is Largely Comparable Between Mononuclear Leukocyte Subsets in PBMCs Isolated by Ficoll-Paque or SepMate Tube Density Gradient Centrifugation

**Figure 3:**
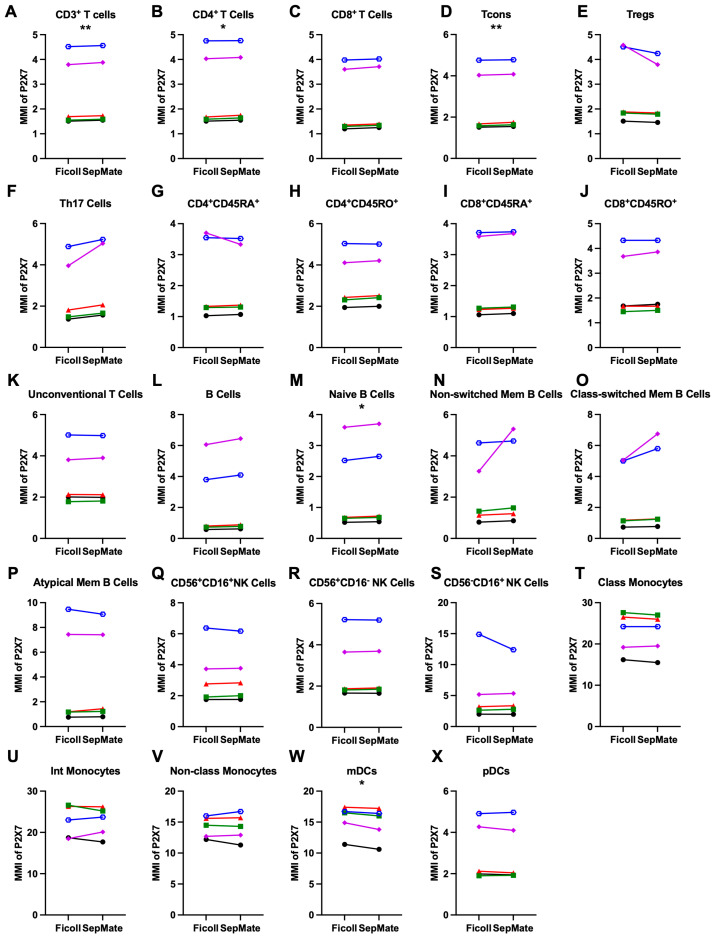
Cell surface P2X7 expression is largely comparable between mononuclear leukocyte subsets in PBMCs isolated by Ficoll-Paque or SepMate Tube density gradient centrifugation. (**A**–**X**) Paired PBMC samples, isolated by Ficoll-Paque (Ficoll) or SepMate Tube (SepMate) density gradient centrifugation, were labelled with metal-conjugated mAbs and analysed by mass cytometry. Sequential flow cytometric gates were selected using a consistent gating strategy as demonstrated in Figure 1. Cell subsets (as per Figure 2) were examined for relative cell surface P2X7 expression. The mean metal intensity (MMI) was determined using the geometric mean function of FlowJo. Symbols represent individual donors (*n* = 5 donors). Statistical analyses were conducted using a (**A**,**B**,**D**,**H**,**J**–**N**,**P**,**R**–**X**) paired Student’s *t*-test or (**C**,**E**–**G**,**I**,**O**,**Q**) Wilcoxon test. * *p* < 0.05; ** *p* < 0.01.

To determine if there were differences in relative cell surface P2X7 expression on mononuclear leukocyte subsets in PBMCs isolated by Ficoll-Paque or SepMate Tube density gradient centrifugation, the above mass cytometric samples were analysed further. P2X7 was present on all PBMC subsets isolated by either separation technique (Figure 3). PBMCs isolated by Ficoll-Paque or SepMate Tube density centrifugation demonstrated a rank order of P2X7 expression (highest to lowest) on classical and intermediate monocytes > non-classical monocytes and mDCs > pDCs, NK cell subsets, T cell subsets, and B cells.

Analysis of T cell subsets showed that P2X7 expression (as the mean of MMI ± SEM for Ficoll-Paque and SepMate Tube, respectively) showed a subtle but significant increase in detection on CD3^+^ T cells (2.6 ± 0.6 and 2.7 ± 0.6) (*p* = 0.008) (Figure 3A), total CD4^+^ T cells (2.7 ± 0.7 and 2.8 ± 0.7) (*p* = 0.01) (Figure 3B), and Tcons (2.7 ± 0.7 and 2.8 ± 0.7) (*p* = 0.008) (Figure 3D) in PBMCs isolated using SepMate Tubes compared to those isolated using Ficoll-Paque. P2X7 expression on total CD8^+^ T cells (2.3 ± 0.6 and 2.3 ± 0.6) (*p* = 0.06) (Figure 3C), Tregs (2.9 ± 0.7 and 2.6 ± 0.6) (*p* = 0.06) (Figure 3E) and Th17 cells (2.7 ± 0.7 and 3.1 ± 0.8) (*p* = 0.06) (Figure 3F) was comparable between separation techniques. Further, naïve CD4^+^CD45RA^+^ T cells (2.2 ± 0.6 and 2.1 ± 0.5) (*p* = 1.0) (Figure 3G), memory CD4^+^CD45RO^+^ T cells (3.2 ± 0.6 and 3.2 ± 0.6) (*p* = 0.06) (Figure 3H), naïve CD8^+^CD45RA^+^ T cells (2.2 ± 0.6 and 2.2 ± 0.6) (*p* = 0.06) (Figure 3I), memory CD8^+^CD45RO^+^ T cells (2.6 ± 0.6 and 2.6 ± 0.6) (*p* = 0.14) (Figure 3J), and unconventional T cells (2.9 ± 0.6 and 3.0 ± 0.6) (*p* = 0.61) (Figure 3K) were comparable between separation techniques.

In regard to B cells, P2X7 expression on total CD19^+^ B cells (2.4 ± 1.1 and 2.6 ± 1.2) (*p* = 0.08) (Figure 3L), as well as non-switched memory B cells (2.2 ± 0.7 and 2.7 ± 1.0) (*p* = 0.28) (Figure 3N), class-switched memory B cells (2.6 ± 1.0 and 3.2 ± 1.3) (*p* = 0.07) (Figure 3O), and atypical memory B cells (4.0 ± 1.8 and 4.0 ± 1.8) (*p* = 0.86) (Figure 3P) was comparable between separation techniques. In contrast, P2X7 expression was slightly but significantly increased on naïve B cells (1.6 ± 0.6 and 1.7 ± 0.6) (*p* = 0.04) (Figure 3M) in PBMCs isolated using SepMate Tubes compared to those isolated by Ficoll-Paque.

In regard to NK cells, P2X7 expression on CD56^+^CD16^+^ NK cells (3.3 ± 0.8 and 3.3 ± 0.8) (*p* = 0.88) (Figure 3Q), CD56^+^CD16^−^ NK cells (2.8 ± 0.7 and 2.9 ± 0.7) (*p* = 0.14) (Figure 3R), and CD56^−^CD16^+^ NK cells (5.6 ± 2.4 and 5.2 ± 1.9) (*p* = 0.81) (Figure 3S) was comparable between separation techniques.

Finally, P2X7 expression on classical monocytes (22.7 ± 2.2 and 22.4 ± 2.2) (*p* = 0.19) (Figure 3T), intermediate monocytes (22.6 ± 1.8 and 22.6 ± 1.6) (*p* = 0.94) (Figure 3U), non-classical monocytes (14.2 ± 0.8 and 14.2 ± 1.0) (*p* = 0.33) (Figure 3V), and pDCs (3.0 ± 0.6 and 3.0 ± 0.6) (*p* = 0.33) (Figure 3X) were comparable between separation techniques. In contrast, P2X7 expression was slightly but significantly decreased on mDCs (15.4 ± 1.1 and 14.8 ± 1.2) (*p* = 0.02) (Figure 3W) in PBMCs isolated using SepMate Tubes compared to those isolated by Ficoll-Paque.

### 3.3. Cell Surface CD39 Expression Is Largely Comparable Between Mononuclear Leukocyte Subsets in PBMCs Isolated by Ficoll-Paque or SepMate Tube Density Gradient Centrifugation

**Figure 4:**
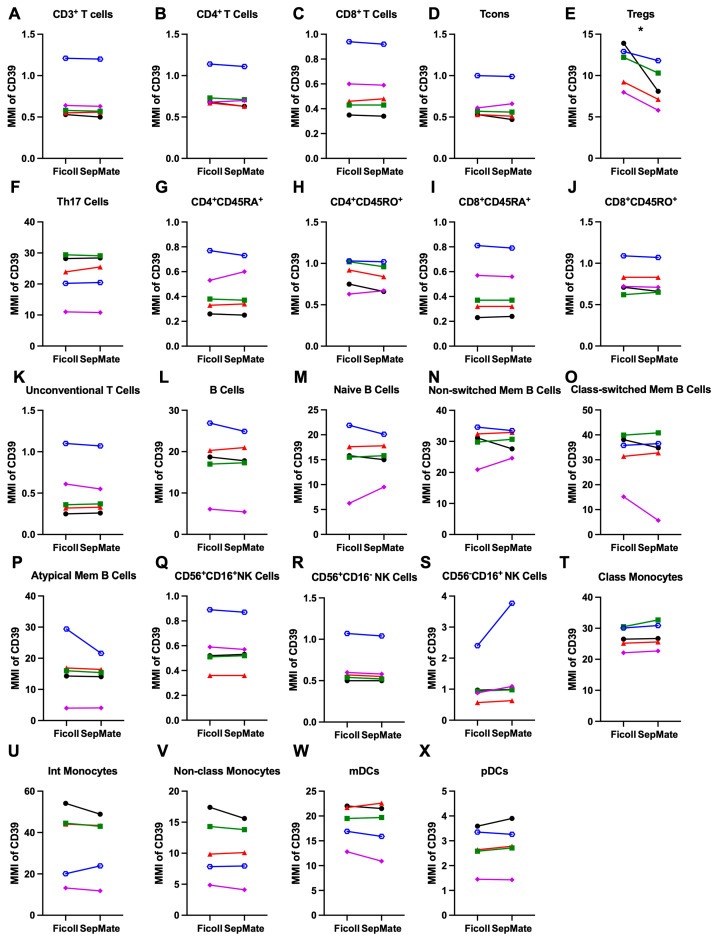
Cell surface CD39 expression is largely comparable between mononuclear leukocyte subsets in PBMCs isolated by Ficoll-Paque or SepMate Tube density gradient centrifugation. (**A**–**X**) Paired PBMC samples, isolated by Ficoll-Paque (Ficoll) or SepMate Tube (SepMate) density gradient centrifugation, were labelled with metal-conjugated mAbs and analysed by mass cytometry. Sequential flow cytometric gates were selected using a consistent gating strategy as demonstrated in Figure 1. Cell subsets (as per Figure 2) were examined for relative cell surface CD39 expression. The mean metal intensity (MMI) was determined using the geometric mean function of FlowJo. Symbols represent individual donors (*n* = 5 donors). Statistical analyses were conducted using a (**A**,**B**,**D**,**O**,**R**,**S**) Wilcoxon or (**C**,**E**–**N**,**P**,**Q**,**T**–**X**) paired Student’s *t*-test with *p* values as shown. * *p* < 0.05.

To determine if there were differences in relative cell surface CD39 expression on mononuclear leukocyte subsets in PBMCs isolated by Ficoll-Paque or SepMate Tube density gradient centrifugation, the above mass cytometric samples were analyzed further. CD39 was present on all PBMC subsets isolated by either separation technique, although relatively low amounts were evident on NK cell subsets and most T cell subsets (Figure 4). PBMCs isolated by Ficoll-Paque or SepMate Tubes demonstrated a rank order of CD39 expression (highest to lowest) on classical and intermediate monocytes > Th17 cells > non-classical monocytes, mDCs and B cells > Tregs > pDCs > NK cell subsets, and the remaining T cell subsets.

Analysis of T cell subsets showed that CD39 expression on CD3^+^ T cells (0.7 ± 0.1 and 0.7 ± 0.1) (*p* = 0.31) (Figure 4A), total CD4^+^ T cells (0.8 ± 0.1 and 0.8 ± 0.1) (*p* = 0.19) (Figure 4B), total CD8^+^ T cells (0.6 ± 0.1 and 0.6 ± 0.1) (*p* = 0.59) (Figure 4C), and Tcons (0.6 ± 0.1 and 0.6 ± 0.1) (*p* = 0.38) (Figure 4D) was negligible but comparable between separation techniques. In contrast, CD39 expression had the most notable and significant reduction on Tregs (11.2 ± 1.1 and 8.6 ± 1.1) (*p* = 0.03) (Figure 4E) in PBMCs isolated using SepMate Tubes compared to Ficoll-Paque isolated cells. In contrast, CD39 expression on Th17 cells (22.5 ± 3.3 and 22.9 ± 3.4) (*p* = 0.40) (Figure 4F), naïve CD4^+^CD45RA^+^ T cells (0.5 ± 0.1 and 0.5 ± 0.1) (*p* = 0.84) (Figure 4G), memory CD4^+^CD45RO^+^ T cells (0.9 ± 0.1 and 0.8 ± 0.1) (*p* = 0.17) (Figure 4H), naïve CD8^+^CD45RA^+^ T cells (0.5 ± 0.1 and 0.5 ± 0.1) (*p* = 0.48) (Figure 4I), memory CD8^+^CD45RO^+^ T cells (0.8 ± 0.1 and 0.8 ± 0.1) (*p* = 0.49) (Figure 4J), and unconventional T cells (0.5 ± 0.2 and 0.5 ± 0.1) (*p* = 0.45) (Figure 4K) was comparable between separation techniques.

In regard to B cells, CD39 was expressed on total CD19^+^ B cells (17.8 ± 3.4 and 17.3 ± 3.3) (*p* = 0.34) (Figure 4L), as well as naïve B cells (15.4 ± 2.6 and 15.6 ± 1.8) (*p* = 0.80) (Figure 4M), non-switched memory B cells (29.8 ± 2.4 and 29.9 ± 1.7) (*p* = 0.94) (Figure 4N), class-switched memory B cells (32.1 ± 4.5 and 30.1 ± 6.3) (*p* = 0.81) (Figure 4O), and atypical memory B cells (16.1 ± 4.0 and 14.3 ± 2.9) (*p* = 0.30) (Figure 4P) was comparable between separation techniques.

In regard to NK cells, CD39 expression on CD56^+^CD16^+^ NK cells (0.6 ± 0.1 and 0.6 ± 0.1) (*p* = 0.59) (Figure 4Q), CD56^+^CD16^−^ NK cells (0.7 ± 0.1 and 0.6 ± 0.1) (*p* = 0.13) (Figure 4R), and CD56^−^CD16^+^ NK cells (1.2 ± 0.3 and 1.5 ± 0.6) (*p* = 0.06) (Figure 4S) was comparable between separation techniques. Notably, CD39 expression on CD56^−^CD16^+^ NK cells from one donor was increased 1.8-fold in PBMCs isolated by SepMate Tubes compared to those isolated by Ficoll-Paque (Figure 4S), despite similar CD39 expression between techniques for the other four donors.

Finally, CD39 expression on classical monocytes (26.9 ± 1.6 and 27.7 ± 1.8) (*p* = 0.08) (Figure 4T), intermediate monocytes (35.2 ± 7.9 and 34.2 ± 7.0) (*p* = 0.52) (Figure 4U), non-classical monocytes (10.9 ± 2.2 and 10.3 ± 2.1) (*p* = 0.21) (Figure 4V), mDCs (18.6 ± 1.7 and 18.1 ± 2.1) (*p* = 0.39) (Figure 4W), and pDCs (2.7 ± 0.4 and 2.8 ± 0.4) (*p* = 0.25) (Figure 4X) was comparable between separation techniques.

## 4. Discussion

The aim of this study was to compare the relative expression of cell surface P2X7 and CD39 on mononuclear leukocyte subsets in human PBMCs isolated by Ficoll-Paque or SepMate Tube density gradient centrifugation. This study indicated that there are minor but statistically significant differences in P2X7 or CD39 expression between some cell subsets in paired PBMC samples isolated by Ficoll-Paque or SepMate Tube density gradient centrifugation. The reasons for the observed differences are unknown but may relate to cell activation which in turn could influence cell surface expression of P2X7 and CD39. A previous comparison of Ficoll-Paque and SepMate Tube isolation methods revealed that human PBMCs isolated using Ficoll-Paque secreted lower amounts of interferon-γ, interleukin (IL)-1β, IL-6, IL-8, and tumour necrosis factor-α compared to PBMCs isolated using SepMate Tubes [20]. Although this prior study did not compare expression of cell surface molecules, cell activation can alter cell surface expression of molecules [28], including P2X7 [29] and CD39 [30].

In addition to general immune activation, the observed differences may reflect isolation-induced alterations in extracellular nucleotide availability during sample processing. Mechanical stress, centrifugation forces, and cell–cell interactions during PBMC isolation are known to promote ATP release, which can in turn modulate the surface expression and function of purinergic receptors and ectonucleotidases [31]. Given that P2X7 and CD39 activity is directly regulated by extracellular ATP concentrations [3,7], even subtle differences in isolation procedures may disproportionately affect these cell surface markers.

In contrast to the P2X7 and CD39 findings, the current study found no significant differences in the proportions of the examined mononuclear leukocyte subsets in PBMCs isolated using SepMate Tubes compared those isolated using Ficoll-Paque. Although comparisons of different isolation techniques by flow cytometry are well documented [20,21,32], evaluations using mass cytometry are scarce. To our knowledge, this is the first study using mass cytometry to compare cell subset composition in PMBCs isolated by SepMate Tube density centrifugation to those isolated by Ficoll-Paque centrifugation. Consistent with the current findings, previous flow cytometric studies have similarly reported no significant differences in the proportions of major lymphocyte subsets between these two isolation methods [20,21,33]. Importantly, the current mass cytometric study extends these earlier findings by demonstrating a comparable composition of monocytes and DCs, as well as lymphocytes in PBMCs, thereby supporting the use of either isolation method in future immunophenotyping studies by either mass or flow cytometry. Future studies are required to compare the effects of other isolation techniques such as apheresis [34] on leukocyte subsets and cell surface molecules.

The findings of this study have implications for clinical and translational research. P2X7 and CD39 are important markers in various diseases, including inflammatory and autoimmune disorders [35,36,37]. Understanding how different isolation methods may affect their expression on immune cells and other cell types is crucial for the interpretation of clinical data and for developing standardised protocols in translational research. The consistency of marker expression is particularly important when these markers are used for diagnostic or prognostic purposes. Further, this could have implications on experiments or disease models utilising human PBMCs. For example, in models of graft-versus-host disease, where both P2X7 and CD39 are involved [38], such differences could affect the interpretation of immune cell function and disease outcomes. Similarly, in cancer immunotherapy research, where both P2X7 and CD39 expression can influence anti-tumour immune responses [39], variations in expression due to isolation methods could influence the evaluation of the efficacy of therapeutic strategies.

This study also showed that PBMCs isolated by Ficoll-Paque or SepMate Tube density gradient centrifugation demonstrated a rank order of cell surface P2X7 expression (highest to lowest) on classical and intermediate monocytes > non-classical monocytes and mDCs > pDCs, NK cell subsets, T cell subsets, and B cells. This data is somewhat comparable to our prior study using the same anti-P2X7 mAb clone and flow cytometry [40]. This prior study revealed a rank order of cell surface P2X7 expression (highest to lowest) on classical and intermediate monocytes > mDCs and pDCs > CD56^−^CD16^+^ NK cells > non-classical monocytes, other NK cell subsets, T cell subsets, and B cells. Differences in the relative expression of P2X7 on non-classical monocytes, pDCs and CD56^−^CD16^+^ NK cells may represent donor differences, use of cryopreserved PBMCs (this study) versus freshly isolated PBMCs (previous study), use of different mAb clones and/or panel design, and subsequent population gating approaches.

Further, this study revealed that PBMCs isolated by Ficoll-Paque or SepMate Tube density gradient centrifugation demonstrated a rank order of cell surface CD39 expression (highest to lowest) on classical and intermediate monocytes > Th17 cells > non-classical monocytes, mDCs and B cells > Tregs > pDCs > NK cell subsets, and various other T cell subsets. This provides the most comprehensive analysis of CD39 expression on PBMCs using mass cytometry reported to date. While the expression of CD39 on PBMCs is well documented by flow cytometry [41], studies reporting CD39 expression using mass cytometry have only documented it on a limited number of PBMC subsets. The cell types reported to express CD39 by mass cytometry include CD4^+^ T cells [42], Tregs [43], B cells [44], and monocytes [45]. It is noteworthy that three of these studies employed the same anti-CD39 mAb clone (clone A1) used in the present study [42,45], indicating reliability of this mAb in mass cytometry. Of note, the relative expression of CD39 on mononuclear subsets overlaps with the relative expression of P2X7 suggesting, that this ectonucleotidase may be present at increased amounts in those cell types with the greatest amounts of cell surface P2X7 to modulate the activation of this receptor.

Finally, this study revealed that for a given cell type, P2X7 or CD39 expression varied between individuals. For P2X7 this may relate to single nucleotide polymorphisms in the *P2RX7* gene, which code for missense mutations, which can affect receptor expression in human mononuclear leukocytes [6]. For CD39, this may also relate to single nucleotide polymorphisms in the *ENTPD1* gene, which can alter CD39 expression; however, such variants are typically intronic and associated with differences between individuals for CD39 expression on T and NK cells, but not B cells or monocytes [46].

In summary, this mass cytometric study reveals comparable amounts of cell surface P2X7 or CD39 on human mononuclear leukocytes in PBMCs isolated by Ficoll-Paque or SepMate Tube density gradient centrifugation. Although small, subset-specific differences were observed, these are unlikely to affect most immunophenotyping studies. Overall, these findings support the use of either isolation method for mass cytometry-based analyses.

## Figures and Tables

**Table 1:** Metal-conjugated anti-human mAbs used for immunolabelling in mass cytometry.

Antibody	Clone	Conjugated Metal
**Barcode**		
CD45	HI30	^106^Pd
CD45	HI30	^110^Pd
**Cocktail**		
CD3	UCHT1	^170^Er
CD4	RPA-T4	^145^Nd
CD8a	RPA-T8	^89^Y
CD11c	Bu15	^115^In
CD14	M5E2	^160^Gd
CD16	B73.1	^148^Nd
CD19	HIB19	^142^Nd
CD25	M-A251	^169^Tm
CD27	M-T271	^167^Er
CD39	A1	^151^Eu
CD45RA	HI100	^143^Nd
CD45RO	UCHL1	^164^Er
CD56	REA196	^176^Lu
CD66b	G10F5	^152^Gd
CD161	DX12	^149^Sm
HLA-DR	L243	^174^Yb
IgD	IA6-2	^146^Nd
P2X7	L4	^141^Pr
**Intracellular**		
FoxP3	PCH101	^162^Er

All mAbs were from commercial suppliers as described [22,24], except for the anti-P2X7 mAb (clone L4) [25,26], which was purified via Protein G chromatography from tissue culture supernatant. All mAbs were conjugated in-house as described [22,24].

## Data Availability

The original contributions presented in this study are included in the article. Further inquiries can be directed to the corresponding author.

## Abbreviations

The following abbreviations are used in this manuscript:

ADP	Adenosine 5′-diphosphate
ATP	Adenosine 5′-triphosphate
DCs	Dendritic cells
FCS	Foetal calf serum
FoxP3	Forkhead box protein P3
IL	Interleukin
mAb	Monoclonal antibody
MMI	Mean metal intensity
NK	Natural killer
PBMCs	Peripheral blood mononuclear cells
RPMI-FCS	RPMI-1640 medium containing 10% FCS
Tcons	Conventional T cells
Th	T helper
Tregs	Regulatory T cells
